# Supplementary material for: A cortical cell ensemble in the posterior parietal cortex controls past experience-dependent memory updating
Source: Nat Commun. 2022 Jan 11;13:41. doi: 10.1038/s41467-021-27763-x (PMC8752845; doi:10.1038/s41467-021-27763-x)
Supplement: Supplementary file 1 — Supplementary information [file 41467_2021_27763_MOESM1_ESM.pdf]

Supplementary Information

A cortical cell ensemble in the posterior parietal cortex controls past experience-dependent memory updating

Akinobu Suzuki[1,2,3], Sakurako Kosugi[1,2], Emi Murayama[1,2,3], Eri Sasakawa[1], Noriaki Ohkawa[1,2,4,6], Ayumu Konno[5], Hirokazu Hirai[5], Kaoru Inokuchi[1,2,3,*]

[1]Department of Biochemistry, Graduate School of Medicine and Pharmaceutical Sciences, University of Toyama, 2630 Sugitani, Toyama 930-0194, Japan
[2]CREST, JST, University of Toyama, Toyama 930-0194, Japan
[3]Research Center for Idling Brain Science, University of Toyama, Toyama 930-0194, Japan
[4]PRESTO, JST, 4-1-8 Honcho, Kawaguchi, Saitama 332-0012, Japan
[5]Department of Neurophysiology and Neural Repair, Gunma University Graduate School of Medicine, Maebashi, Gunma, 371-8511, Japan
[6]Present address: Division for Memory and Cognitive Function, Research Center for Advanced Medical Science, Comprehensive Research Facilities for Advanced Medical Science, Dokkyo Medical University, Tochigi 321-0293, Japan

*Corresponding Author, Email: inokuchi@med.u-toyama.ac.jp

Supplementary Figures

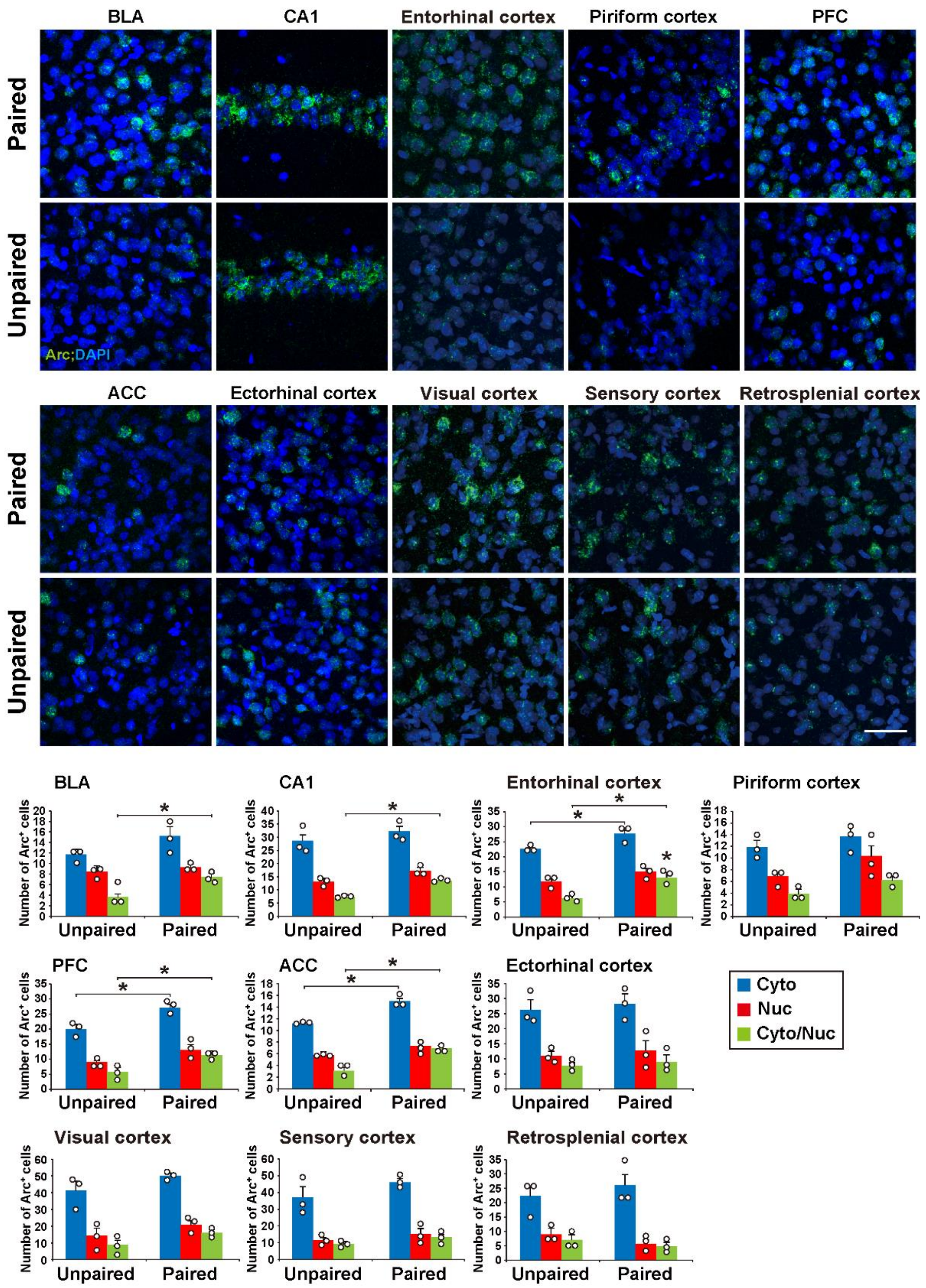

Supplementary Fig. 1. Multiple brain regions respond to the pre-exposed context and IS.

Representative images of the Arc CatFISH analysis in several brain regions (top). The Arc RNA signal and DAPI nuclear staining are shown in green and blue, respectively.

Scale bar, 50 μm. Number of Arc+ cells (bottom) (n = 3 mice/group) (Two-tailed Unpaired t-test, BLA; Cyto: $P = 0.1464$, Nuc: $P = 0.3791$, Cyto/Nuc: $P = 0.0058$, CA1; Cyto: $P = 0.3701$, Nuc: $P = 0.1121$, Cyto/Nuc: $P = 0.0002$, Entorhinal cortex; Cyto: $P = 0.0383$, Nuc: $P = 0.0624$, Cyto/Nuc: $P = 0.0059$, Piriform cortex; Cyto: $P = 0.3813$, Nuc: $P = 0.1680$, Cyto/Nuc: $P = 0.0522$, PFC; Cyto: $P = 0.0223$, Nuc: $P = 0.0731$, Cyto/Nuc: $P = 0.0108$, ACC; Cyto: $P = 0.0027$, Nuc: $P = 0.0866$, Cyto/Nuc: $P = 0.0039$, Ectorhinal cortex; Cyto: $P = 0.6870$, Nuc: $P = 0.6429$, Cyto/Nuc: $P = 0.5995$, Visual cortex; Cyto: $P = 0.2168$, Nuc: $P = 0.3354$, Cyto/Nuc: $P = 0.0979$, Sensory cortex; Cyto: $P = 0.1992$, Nuc: $P = 0.3591$, Cyto/Nuc: $P = 0.1897$, Retrosplenial cortex; Cyto: $P = 0.5966$, Nuc: $P = 0.1861$, Cyto/Nuc: $P = 0.3285$). Error bars indicate the mean ± s.e.m. $^*P < 0.05$. For details of statistical data, see Supplementary Table 2. Cyto, cytoplasmic; Nuc, nuclear. Source data are provided as a Source Data file.

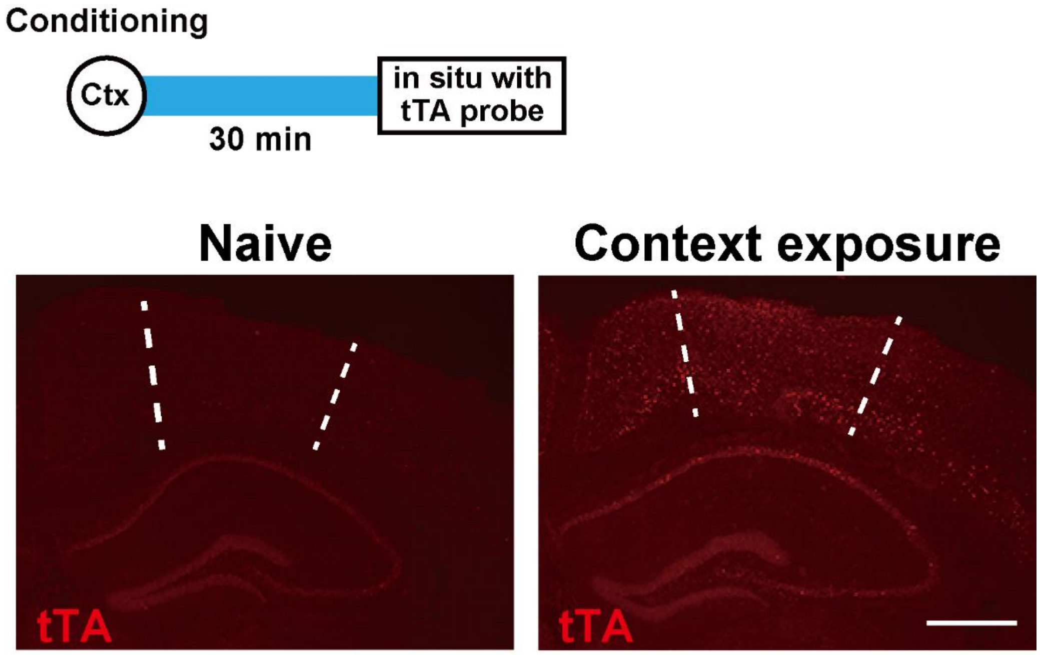

Supplementary Fig. 2. tTA mRNA is expressed in the PPC.

Schema of the behavioral and in situ experiments. Mice were sacrificed 30 min after context exposure (top). Control mice without context exposure were used as naïve mice. Representative images of the tTA in situ analysis in naïve and context-exposed mice (bottom). These immunohistochemical analyses were repeated at least two times independently with similar results. Red dots represent the tTA mRNA signal. Broken lines indicate the PPC boundaries. Scale bar, 500 μm. Ctx, context.

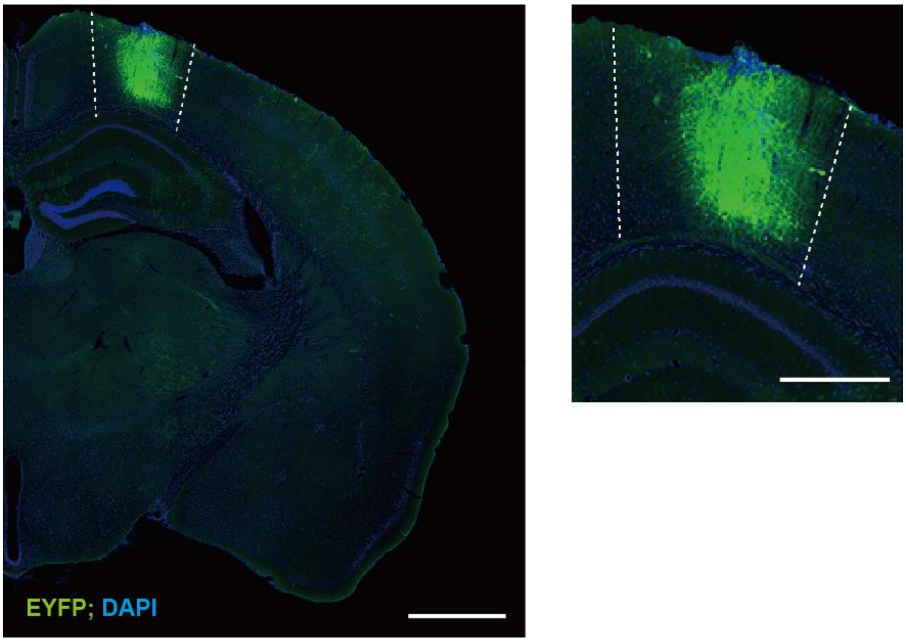

Supplementary Fig. 3. Expression of ArchT-EYFP in the PPC.

Representative labeling pattern of PPC cells with ArchT-EYFP protein in a c-fos-tTA transgenic mice that was conditioned with context exposure at 2 days after OFF Dox (left). The signal of fluorescent protein and DAPI nuclear staining at 1 day after the conditioning are shown in green and blue, respectively. This section was taken from bregma -1.94 mm. Scale bar, 1 mm. An enlarged image of the injection area (right). These immunohistochemical analyses were repeated at least three times independently with similar results. Broken lines indicate the PPC boundaries. Scale bar, 500 μm.

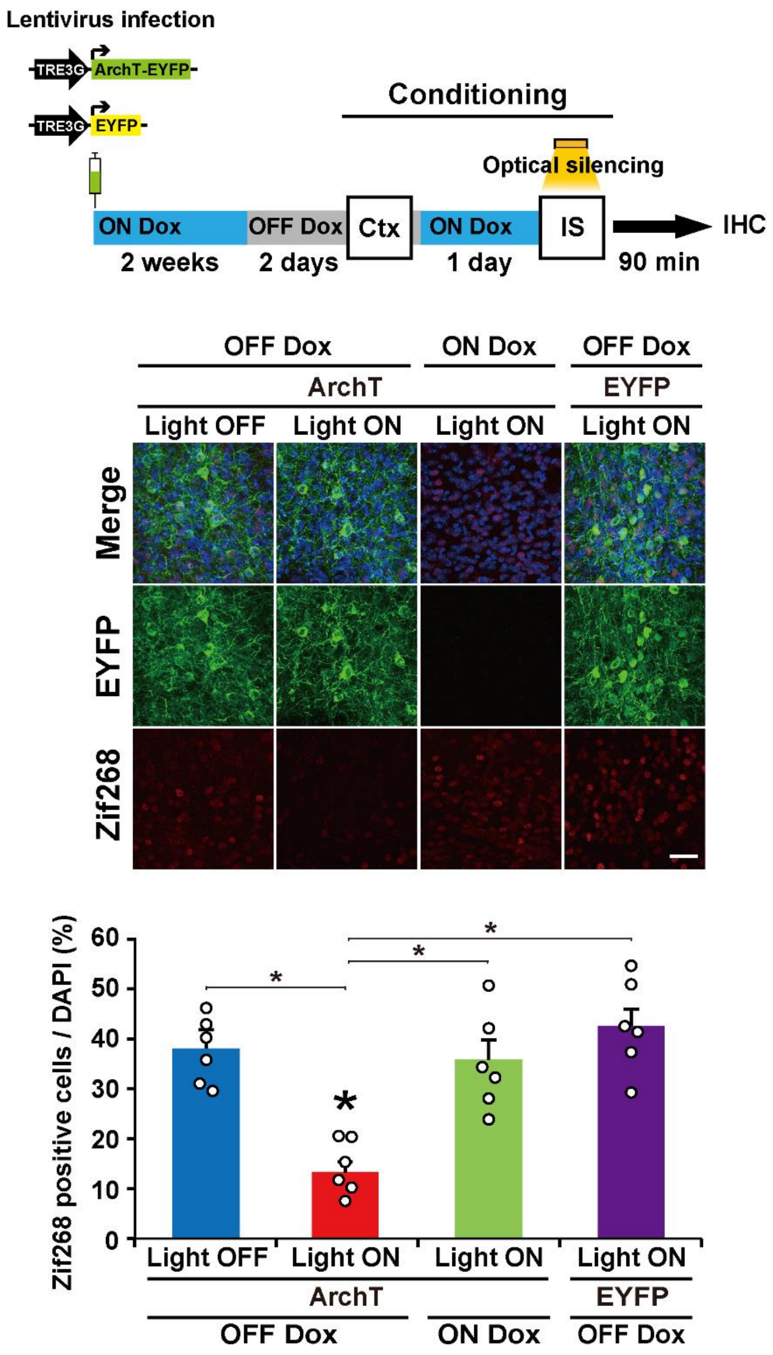

Supplementary Fig. 4. ArchT-EYFP functions in the Light ON and OFF Dox condition.

The behavioral experiment with optical silencing (top). Blue and gray bars indicate the presence or absence of Dox, respectively. A representative image of Zif268 expression 90 min after an IS session with or without optical silencing (middle). Scale bar, 100 μm. The proportion of Zif268-positive cells in each group (bottom) (n = 6 sections from 2 mice/group) (One-way ANOVA followed by Tukey's post hoc tests, $P < 0.0001$). Error

bars indicate the mean ± s.e.m. *P < 0.05. For details of statistical data, see Supplementary Table 2. Source data are provided as a Source Data file.

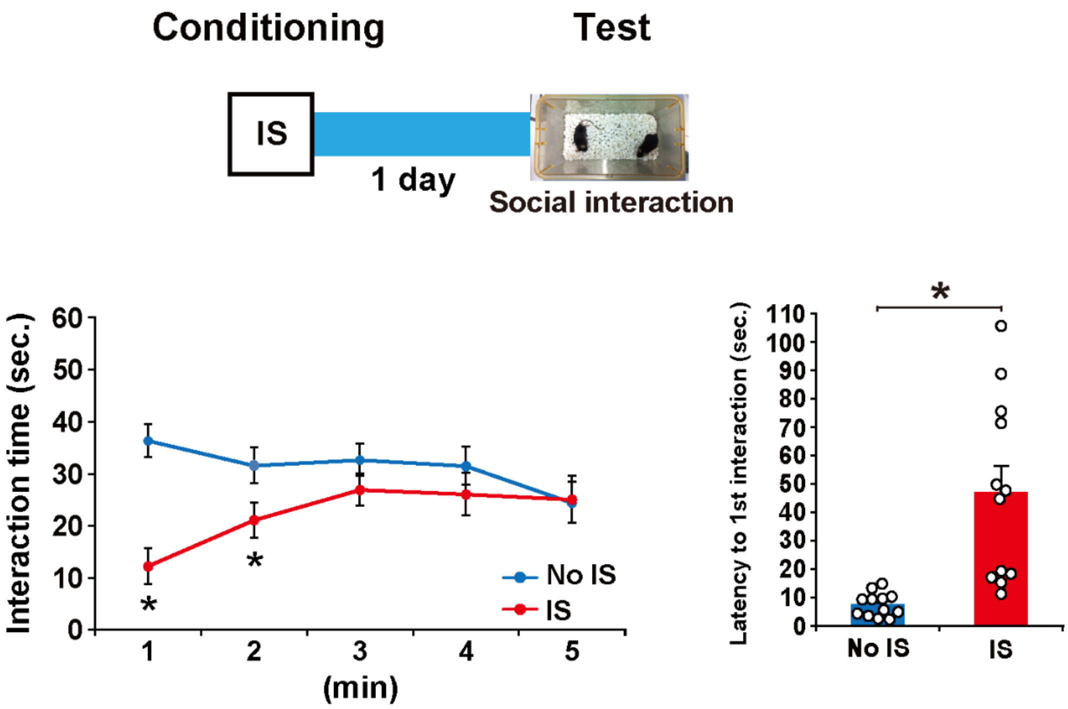

Supplementary Fig. 5. IS affects the social interaction.

Schematic of the behavioral experiment (top). Graph shows the interaction time (bottom left) and the latency to the first interaction (bottom right) during the test (bottom) (No-IS, n = 12 mice; IS, n = 12 mice) (Interaction time; Two-way ANOVA with RM followed by Sidak's post hoc test, time: P = 0.4572, group: P = 0.0001, time x group: P = 0.0122, Latency to 1st interaction; Two-tailed Unpaired t-test, P = 0.0003). Error bars indicate the mean ± s.e.m. *P, < 0.05. IS, immediate shock. For details of statistical data, see Supplementary Table 2. Source data are provided as a Source Data file.

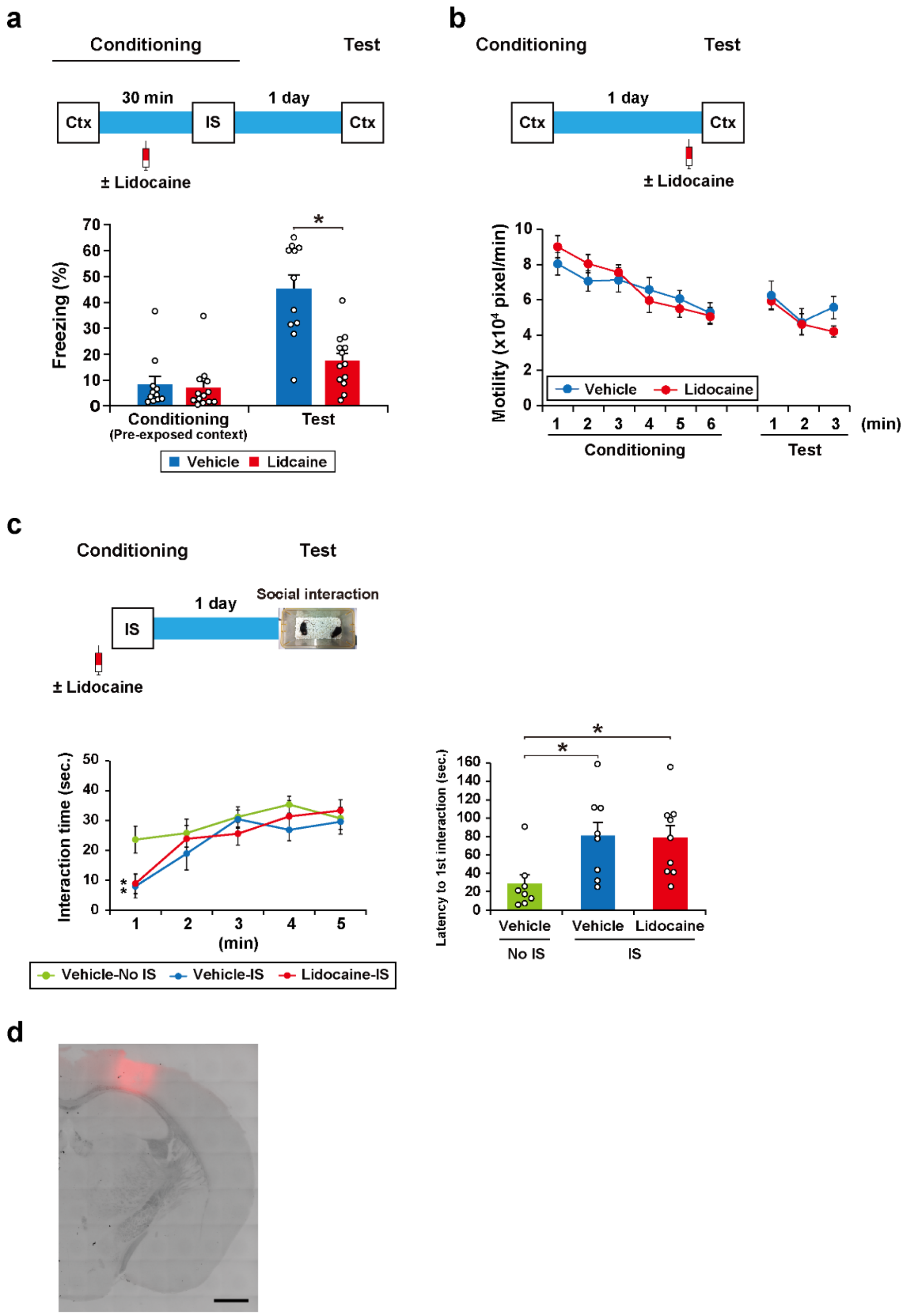

Supplementary Fig. 6. Lidocaine injection in the PPC blocks memory association.
a, Schematic of the behavioral experiment (top). The mice were pre-exposed to context B for 6 min, and then received IS in the same context at intervals of 30 min. The sodium channel blocker lidocaine was injected into the PPC 15 min before IS exposure. The effect of the lidocaine injection on memory association is shown (bottom). The graph shows the freezing level during the conditioning and test session (vehicle, n = 11 mice, lidocaine, n

= 13 mice) (Two-way ANOVA with RM followed by Sidak's post hoc test, session: P < 0.0001, group: P = 0.0016, session x group: P = 0.0004). **b**, Schematic of the behavioral experiment (top). The graph shows motility at the conditioning and test session (bottom) (vehicle, n = 7 mice, lidocaine, n = 8 mice) (Two-way ANOVA with RM followed by Sidak's post hoc test, Conditioning; time: P < 0.0001, group: P = 0.6077, time x group: P = 0.6094, Test; time: P = 0.0643, group: P = 0.2469, time x group: P = 0.5894). **c**, Schematic of the behavioral experiment (top). The graph shows the interaction time (left) and the latency to the first interaction (right) during the test session (bottom) (vehicle-No IS, n = 8 mice, vehicle-IS, n = 8 mice, lidocaine-IS (+), n = 9 mice) (Interaction time; Two-way ANOVA with RM followed by Sidak's post hoc test, time: P < 0.0001, group: P = 0.2434, time x group: P = 0.2314, Latency to 1st interaction; One-way ANOVA followed by Tukey's post hoc tests, P = 0.0210). Error bars indicate the mean ± s.e.m. *P < 0.05. **d**, Representative image of a rhodamine injection into the PPC. All mice were injected with rhodamine in the PPC through a guide cannula to check the coordinates after the behavioral experiments. Scale bar, 1 mm. Ctx, context; IS, immediate shock. For details of statistical data, see Supplementary Table 2. Source data are provided as a Source Data file.

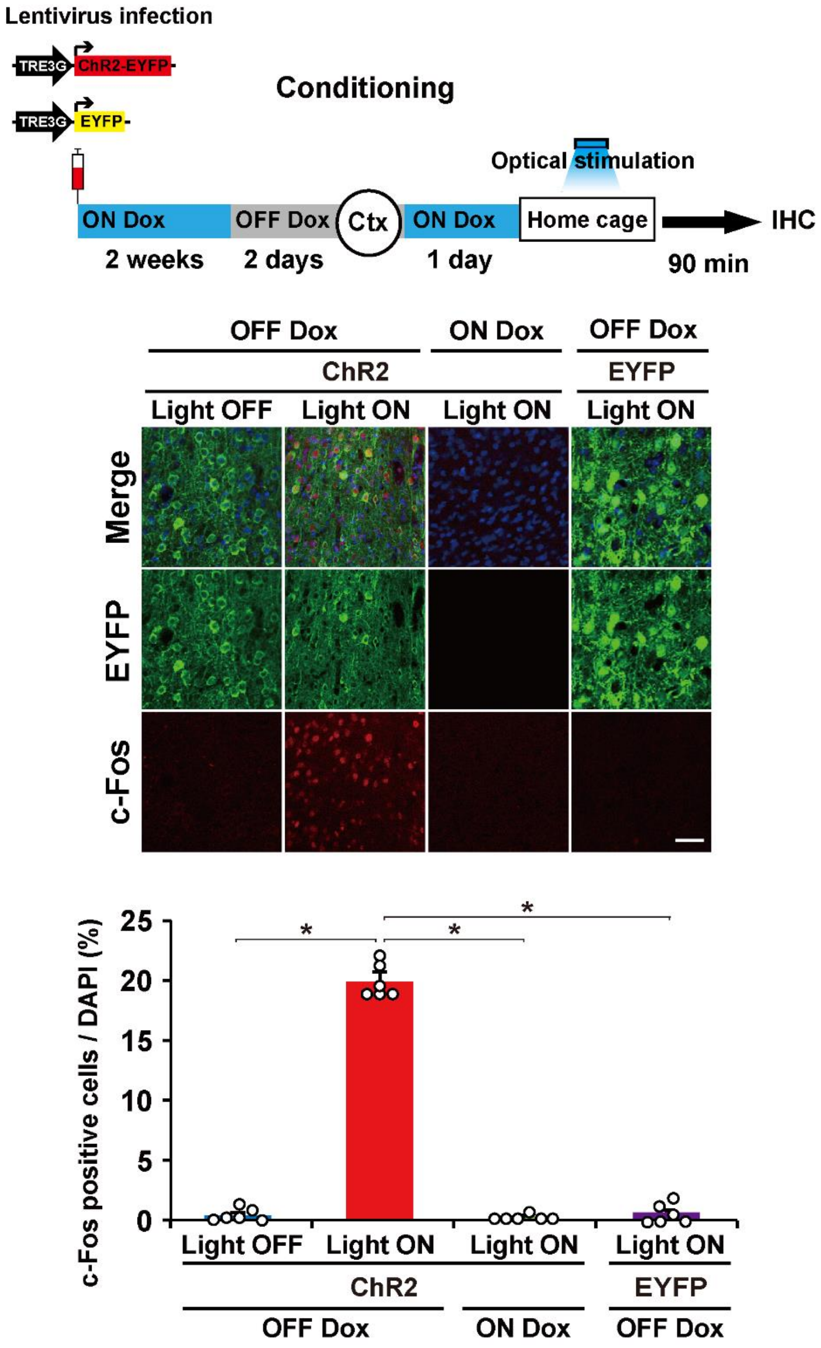

Supplementary Fig.7. ChR2-EYFP functions in the Light ON and OFF Dox condition.

The behavioral experiment with optical stimulation (top). Blue and gray bars indicate the presence or absence of Dox, respectively. Mice were sacrificed 90 min after optical stimulation. A representative image of c-Fos expression (middle). Scale bar, 100 μm. The proportion of c-Fos-positive cells in each group (bottom) (n = 6 sections from 2 mice/group) (One-way ANOVA followed by Tukey's post hoc tests, $P < 0.0001$). Error

bars indicate the mean ± s.e.m. *P < 0.05. For details of statistical data, see Supplementary Table 2. Source data are provided as a Source Data file.

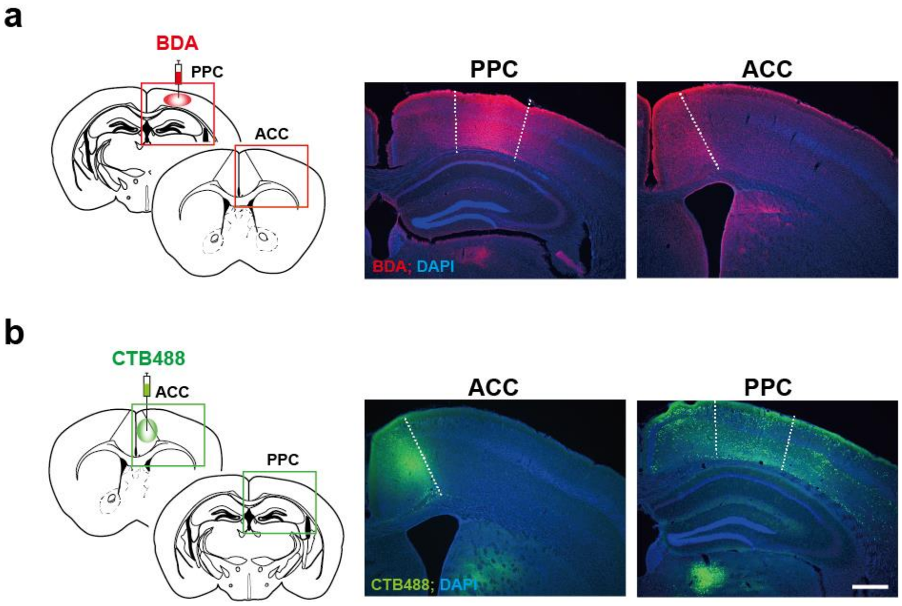

Supplementary Fig. 8. PPC neurons project to the ACC.

a, Schematics showing the BDA injection area and observational area (left). BDA was injected into the PPC. Magnified images of the red square areas are shown on the right. Representative images of brain sections of the PPC and ACC immunostained with the streptavidin-Alexa Fluor 555 (red, right) **b**, Schematics showing the CTB488 injection area and observational area (left). CTB488 was injected into the ACC. Magnified images of the green square areas are shown on the right. Representative images of ACC and PPC brain sections are shown (right). These immunohistochemical analyses were repeated at least three times independently with similar results. Scale bar, 500 μm.

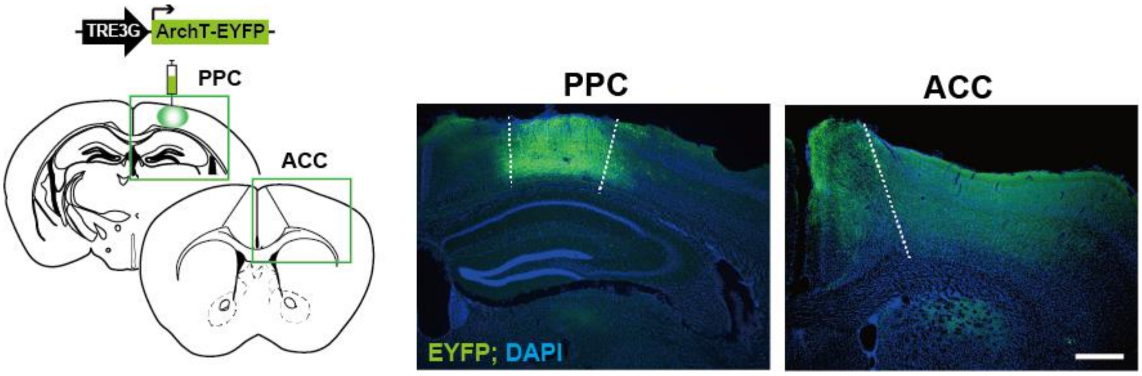

Supplementary Fig. 9. Expression of ArchT-EYFP in neurons in the PPC and PPC axons in the ACC.

Schematics showing labeling of the PPC neurons in c-fos-tTA transgenic mice with the AAV9-TRE3G-ArchT-EYFP (left). Representative images of PPC and ACC brain sections are shown (right). These immunohistochemical analyses were repeated at least three times independently with similar results. Scale bar, 500 μm.

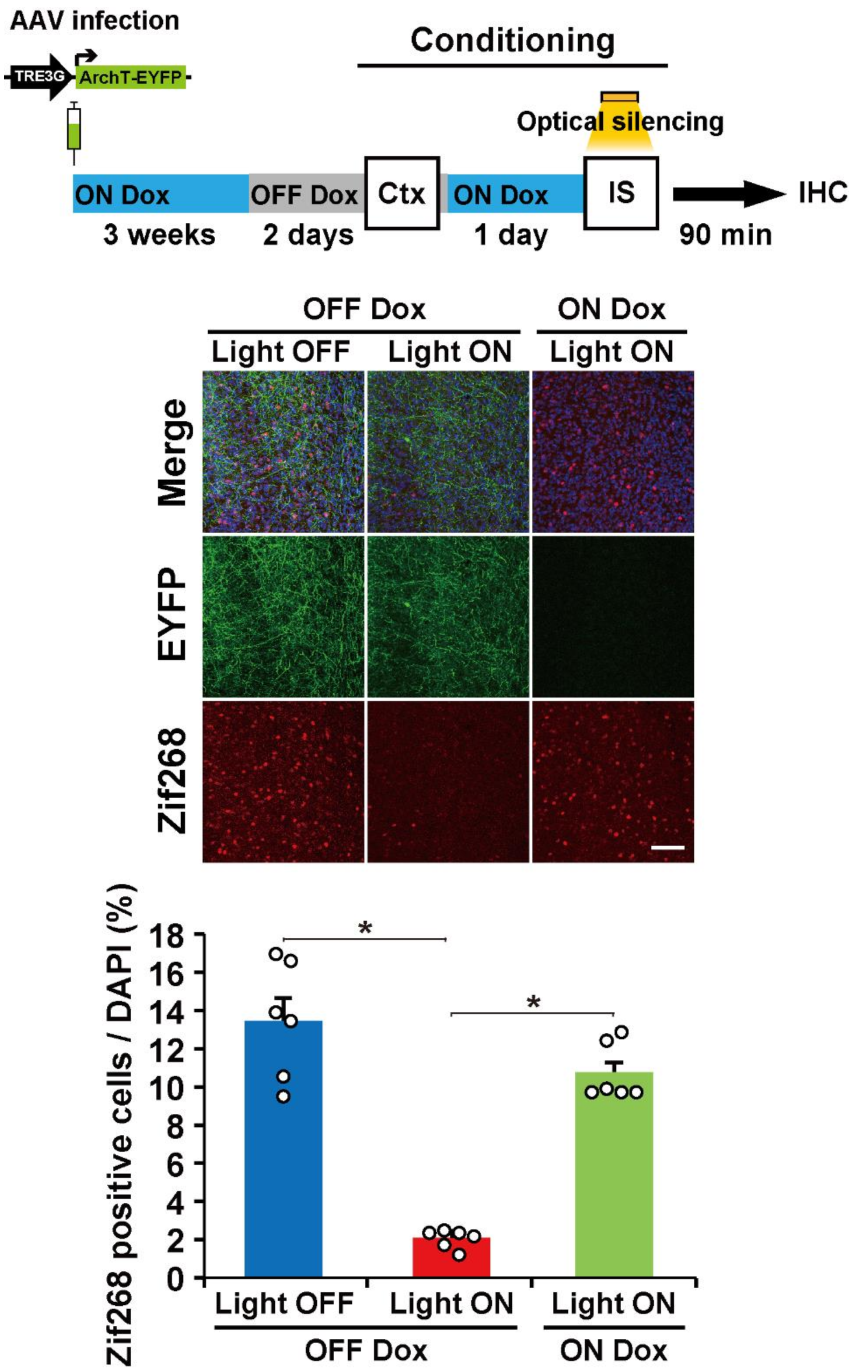

Supplementary Fig. 10. ArchT-EYFP in ACC functions in the Light ON and OFF Dox condition.

The behavioral experiment with optical silencing (top). Blue and gray bars indicate the presence or absence of Dox, respectively. Optical silencing to the ACC was delivered during an IS session of conditioning. A representative image of Zif268 expression 90 min after the IS session with or without optical silencing (middle). Scale bar, 100 μm. The proportion of Zif268-positive cells in each group (bottom) (n = 6 sections from 2

mice/group) (One-way ANOVA followed by Tukey's post hoc tests, $P < 0.0001$). Error bars indicate the mean ± s.e.m. *$P < 0.05$. For details of statistical data, see Supplementary Table 2. Source data are provided as a Source Data file.

Supplementary Table 1. Sampling and statistical analysis details in Fig. 1 to Fig. 7.

Fig. #	Category	Group	Sample size (n)		Score	Statistical test	Degree of Freedom & F/t Value	p-value	Significance?
			Exact size (n)	Excluded (n)					
1b	Freezing (%) in Conditioning	PEC	9	0	3.6 ± 1.22%	Two-tailed unpaired t-test	t16 = 0.2515	P = 0.8047	N.S.
		IS	9	0					
		Paired	9	0	3.9 ± 1.13%				
	Freezing (%) in Test	PEC	9	0	4.6 ± 1.14%	One-way ANOVA	F2,24 = 59.96	P < 0.0001	****
		IS	9	0	8.8 ± 2.45%				
		Paired	9	0	33.1 ± 4.68%				
1c	Freezing (%)	30 min interval Unpaired	8	0	Conditioning; 16.4 ± 2.26%, Test; 16.1 ± 3.79%	Two-way ANOVA with RM	session; F1,14 = 40.44<br>group; F1,14 = 6.596<br>session x group; F1,14 = 41.58	session; P < 0.0001<br>group; P = 0.0223<br>session x group; P < 0.0001	session; ****<br>group; *<br>session x group; ****
		30 min interval Paired	8	0	Conditioning; 10.9 ± 2.99%, Test; 45.0 ± 5.23%				
		1 day interval Unpaired	8	0	Conditioning; 12.1 ± 3.21%, Test; 13.9 ± 3.80%	Two-way ANOVA with RM	session; F1,14 = 22.47<br>group; F1,14 = 6.741<br>session x group; F1,14 = 17.95	session; P = 0.0003<br>group; P = 0.0211<br>session x group; P = 0.0008	session; ***<br>group; *<br>session x group; ***
		1 day interval Paired	8	0	Conditioning; 8.9 ± 3.57%, Test; 41.2 ± 5.63%				
1f	Arc+ cells / DAPI (%)	Unpaired	6	0	Cyto; 28.9 ± 3.24%, Nuc; 5.5 ± 0.57%, Cyto/Nuc; 3.5 ± 0.24%	One-way ANOVA	Cytoplasmic arc; F2,12 = 3.837<br>Nuclear arc; F2,12 = 20.38<br>Cytoplasmic and nuclear arc; F2,12 = 26.95	Cytoplasmic arc; P = 0.0515<br>Nuclear arc; P = 0.0001<br>Cytoplasmic and nuclear arc; P < 0.0001	Cytoplasmic arc; N.S.<br>Nuclear arc; ***<br>Cytoplasmic and nuclear arc; ****
		Paired	6	0	Cyto; 39.3 ± 2.11%, Nuc; 12.6 ± 0.96%, Cyto/Nuc; 10.5 ± 0.98%	Two-tailed unpaired t-test (for vs Chance)	Unpaired; t10 = 4.462<br>Paired; t10= 4.796<br>No IS; t4 = 3.344	Unpaired; P= 0.0012<br>Paired; P= 0.0007<br>No IS; P= 0.0287	Unpaired; **<br>Paired; ***<br>No IS; *
		No IS	3	0	Cyto; 35.6 ± 3.36%, Nuc; 7.5 ± 1.21%, Cyto/Nuc; 5.6 ± 0.78%				
2b	Freezing (%)	ArchT-Light OFF	9	0	Conditioning; 8.1 ± 1.74%, Test; 44.9 ± 4.01%	Two-way ANOVA with RM	session; F1,29 = 147.1<br>group; F2,29 = 5.579<br>session x group; F2,29 = 7.879	session; P < 0.0001<br>group; P = 0.0089<br>session x group; P = 0.0018	session; ****<br>group; **<br>session x group; **
		ArchT-Light ON	11	0	Conditioning; 9.5 ± 2.23%, Test; 24.6 ± 3.99%				
		EYFP-Light ON	12	0	Conditioning; 12.0 ± 2.69%, Test; 41.3 ± 2.90%				
2c	Freezing (%)	Circle labeled	12	0	Conditioning (Circle-context); 4.7 ± 0.66%, Conditioning (Square-context); 7.9 ± 1.59%,<br>Test; 44.1 ± 4.81%	Two-way ANOVA with RM	session; F2,44 = 131.0<br>group; F1,22 = 14.78<br>session x group; F2,44 = 16.64	session; P < 0.0001<br>group; P = 0.0009<br>session x group; P < 0.0001	session; ****<br>group; ***<br>session x group; ****
		Square labeled	12	0	Conditioning Circle-context; 5.5 ± 0.97%, Conditioning Square-context; 6.0 ± 0.99%,<br>Test; 23.8 ± 2.30%				
2d	Motility (pixel/min) Target: PPC	ArchT-Light OFF	10	0	Conditioning;<br>1 min; 86846 ± 5272 pixel, 2 min; 70844 ± 6725 pixel, 3 min; 56555 ± 4866 pixel,<br>4 min; 45718 ± 3409 pixel, 5 min; 45532 ± 2982 pixel, 6 min; 46405 ± 4473 pixel,<br><br>Test;<br>1 min; 33327 ± 5531 pixel, 2 min; 26145 ± 3957 pixel, 3 min; 33446 ± 4277 pixel	Two-way ANOVA with RM	Conditioning Session;<br>time; F5,144 = 34.44<br>group; F2,144 = 1.168<br>time x group; F10,144 = 0.4690<br><br>Test session;<br>time; F2,72 = 5.013<br>group; F2,72 = 0.841<br>time x group; F4,72 = 0.7140	Conditioning Session;<br>time; P < 0.0001<br>group; P = 0.3140<br>time x group; P = 0.9077<br><br>Test session;<br>time; P = 0.0092<br>group; P = 0.4356<br>time x group; P = 0.5851	Conditioning Session;<br>time; ****<br>group; N.S.<br>time x group; N.S.<br><br>Test session;<br>time; **<br>group; N.S.<br>time x group; N.S.
		ArchT-Light ON	10	0	Conditioning;<br>1 min; 95929 ± 8908 pixel, 2 min; 70113 ± 6550 pixel, 3 min; 52386 ± 5710 pixel,<br>4 min; 41445 ± 3653 pixel, 5 min; 41623 ± 3577 pixel, 6 min; 38405 ± 2741 pixel,<br><br>Test;<br>1 min; 42736 ± 3870 pixel, 2 min; 29996 ± 4097 pixel, 3 min; 30547 ± 4790 pixel				
		EYFP-Light ON	7	0	Conditioning;<br>1 min; 92270 ± 6524 pixel, 2 min; 70335 ± 6794 pixel, 3 min; 59980 ± 7856 pixel,<br>4 min; 54518 ± 6741 pixel, 5 min; 47799 ± 3695 pixel, 6 min; 45413 ± 4996 pixel,<br><br>Test;<br>1 min; 39571 ± 6976 pixel, 2 min; 23830 ± 5477 pixel, 3 min; 25186 ± 2771 pixel				
	Motility (pixel/min) Target: CA1	ArchT-Light OFF	12	0	Conditioning;<br>1 min; 87004 ± 5881 pixel, 2 min; 71926 ± 5358 pixel, 3 min; 69915 ± 4195 pixel,<br>4 min; 59864 ± 3278 pixel, 5 min; 54537 ± 2559 pixel, 6 min; 46741 ± 2672 pixel<br><br>Test;<br>1 min; 36302 ± 4323 pixel, 2 min; 29475 ± 5473 pixel, 3 min; 32384 ± 4696 pixel	Two-way ANOVA with RM	Conditioning session;<br>time; F5,204 = 27.87<br>group; F2,204 = 0.9729<br>time x group; F10,204 = 0.4125<br><br>Test session;<br>time; F2,102 = 1.457,<br>group; F2,102 = 20.62,<br>time x group; F4,102 = 0.3644	Conditioning session;<br>time; P < 0.0001<br>group; P = 0.3797<br>time x group; P = 0.9397<br><br>Test session;<br>time; P = 0.2378<br>group; P < 0.0001<br>time x group; P = 0.8334	Conditioning session;<br>time; ****<br>group; N.S.<br>time x group; N.S.<br><br>Test session;<br>time; N.S.<br>group; ****<br>time x group; N.S.
		ArchT-Light ON	12	0	Conditioning;<br>1 min; 86810 ± 4991 pixel, 2 min; 67213 ± 5479 pixel, 3 min; 66419 ± 4554 pixel,<br>4 min; 48235 ± 5903 pixel, 5 min; 55877 ± 5208 pixel, 6 min; 47262 ± 6633 pixel<br><br>Test;<br>1 min; 66052 ± 5315 pixel, 2 min; 59284 ± 6265 pixel, 3 min; 52966 ± 7283 pixel				
		EYFP-Light ON	13	0	Conditioning;<br>1 min; 90508 ± 3722 pixel, 2 min; 68503 ± 4718 pixel, 3 min; 74264 ± 4500 pixel,<br>4 min; 55114 ± 5655 pixel, 5 min; 53087 ± 4934 pixel, 6 min; 51912 ± 3699 pixel<br><br>Test;<br>1 min; 40207 ± 5468 pixel, 2 min; 34212 ± 4692 pixel, 3 min; 37770 ± 4492 pixel				
2e	Interaction time (sec.) Target: PPC	ArchT-Light OFF	10	0	1 min: 5.7 ± 3.23 sec, 2 min; 24.3 ± 4.95 sec, 3 min; 24.1 ± 3.97 sec,<br>4 min; 29.7 ± 5.36 sec, 5 min; 29.3 ± 6.78 sec	Two-way ANOVA with RM	time; F4,120 = 11.52<br>group; F2,120 = 0.5590<br>time x group; F8,120 = 0.4154	time; P < 0.0001<br>group; P = 0.5733<br>time x group; P = 0.9098	time; ****<br>group; N.S.<br>time x group; N.S.
		ArchT-Light ON	10	0	1 min: 10.2 ± 3.81 sec, 2 min; 21.1 ± 5.86 sec, 3 min; 26.8 ± 6.65 sec,<br>4 min; 33.6 ± 4.07 sec, 5 min; 35.8 ± 4.03 sec				
		EYFP-Light ON	7	0	1 min: 5.3 ± 4.58 sec, 2 min; 16.3 ± 5.41 sec, 3 min; 30.3 ± 4.14 sec,<br>4 min; 34.3 ± 6.38 sec, 5 min; 26.4 ± 6.37 sec				
	Latency to 1st interaction (sec.) Target: PPC	ArchT-Light OFF	10	0	93.3 ± 24.96 sec	One-way ANOVA	F2,24 = 0.008	P = 0.9920	N.S.
		ArchT-Light ON	10	0	90.0 ± 21.04 sec				
		EYFP-Light ON	7	0	93.7 ± 20.35 sec				

	Interaction time (sec.) Target: BLA	ArchT-Light OFF	11	0	1 min; 11.3 ± 5.08 sec, 2 min; 23.7 ± 5.82 sec, 3 min; 29.8 ± 6.12 sec,<br>4 min; 34.7 ± 4.27 sec, 5 min; 31.2 ± 4.65 sec	Two-way ANOVA with RM	time; $F_{4,128}$ = 1.437<br>group; $F_{4,128}$ = 12.05<br>time x group; $F_{2,32}$ = 1.080	time; P = 0.1873<br>group; P < 0.0001<br>time x group; P = 0.3515	time; N.S.<br>group; ****<br>time x group; N.S.
		ArchT-Light ON	12	0	1 min; 25.3 ± 3.44 sec, 2 min; 30.3 ± 3.45 sec, 3 min; 35.9 ± 2.83 sec,<br>4 min; 34.5 ± 2.46 sec, 5 min; 25.3 ± 3.62 sec				
		EYFP-Light ON	12	0	1 min; 9.9 ± 4.03 sec, 2 min; 25.5 ± 4.31 sec, 3 min; 29.8 ± 3.92 sec,<br>4 min; 32.1 ± 3.81 sec, 5 min; 28.8 ± 3.11 sec				
	Latency to 1st interaction (sec.) Target: BLA	ArchT-Light OFF	11	0	94.4 ± 25.88 sec	One-way ANOVA	$F_{2,32}$ = 4.389	P = 0.00207	**
		ArchT-Light ON	12	0	34.1 ± 7.88 sec				
		EYFP-Light ON	12	0	97.3 ± 14.60 sec				
3b	Freezing (%)	ChR2-Light OFF in circle	17	0	Conditioning; 9.5 ± 2.37%, Test; 15.6 ± 2.43%	Two-way ANOVA with RM	session; $F_{1,53}$ = 28.02<br>group; $F_{4,53}$ = 12.53<br>session x group; $F_{4,53}$ = 18.19	session; P < 0.0001<br>group; P < 0.0001<br>session x group; P < 0.0001	session; ****<br>group; ****<br>session x group; ****
		ChR2-Light ON in circle	19	0	Conditioning; 8.6 ± 2.07%, Test; 36.4 ± 4.08%				
		EYFP-Light ON in circle	9	0	Conditioning; 8.6 ± 2.17%, Test; 11.2 ± 2.23%				
		ChR2-Light OFF in triangle	7	0	Conditioning; 10.4 ± 4.88%, Test; 7.2 ± 2.70%				
		ChR2-Light ON in triangle	6	0	Conditioning; 4.5 ± 2.36%, Test; 11.4 ± 3.59%				
4b	Freezing (%)	ArchT-Light OFF	14	0	Conditioning; 10.1 ± 2.95%, Reactivation; 44.5 ± 3.87%, Test 1; 48.4 ± 2.38%, Test 2; 44.2 ± 2.22%	Two-way ANOVA with RM	session; $F_{3,102}$ = 76.30<br>group; $F_{2,34}$ = 0.4491<br>session x group; $F_{6,102}$ = 4.863	session; P < 0.0001<br>group; P = 0.6419<br>session x group; P = 0.0002	session; ****<br>group; N.S.<br>session x group; ***
		ArchT-Light ON	13	0	Conditioning; 14.7 ± 3.72%, Reactivation; 46.9 ± 3.73%, Test 1; 28.5 ± 4.18%, Test 2; 50.5 ± 2.52%				
		EYFP-Light ON	10	0	Conditioning; 9.9 ± 2.29%, Reactivation; 40.9 ± 3.89%, Test 1; 43.8 ± 4.14%, Test 2; 42.0 ± 3.83%				
5b	Freezing (%)	ArchT-Light OFF	11	4	Conditioning; 8.1 ± 1.33%, Reactivation; 45.2 ± 4.40%, Test 1; 50.7 ± 4.04%, Test 2; 49.9 ± 2.95%	Two-way ANOVA with RM	session; $F_{3,96}$ = 87.92<br>group; $F_{2,32}$ = 2.549<br>session x group; $F_{6,96}$ = 4.245	session; P < 0.0001<br>group; P = 0.0939<br>session x group; P = 0.0008	session; ****<br>group; N.S.<br>session x group; ***
		ArchT-Light ON	11	4	Conditioning; 9.6 ± 1.91%, Reactivation; 39.9 ± 2.27%, Test 1; 50.1 ± 4.20%, Test 2; 29.4 ± 4.02%				
		EYFP-Light ON	13	0	Conditioning; 9.9 ± 1.54%, Reactivation; 43.9 ±4.61%, Test 1; 41.3 ± 4.39%, Test 2; 50.0 ± 2.53%				
	Freezing (%) in Test 2	ArchT-Light OFF	11	4	49.9 ± 3.70%	One-way ANOVA	$F_{2,32}$ = 13.64	P < 0.0001	****
		ArchT-Light ON	11	4	29.4 ± 5.04%				
		EYFP-Light ON	13	0	50.0 ± 3.45%				
5c	Freezing (%)	Vehicle in CA1	16	0	Conditioning; 13.9 ± 1.66%, Reactivation; 50.0 ± 2.38%, Test 1; 51.6 ± 2.29%, Test 2; 22.1 ± 2.21%	Two-way ANOVA with RM	session; $F_{3,93}$ = 157.6<br>group; $F_{1,31}$ = 0.9200<br>session x group; $F_{3,93}$ = 0.8081	session; P < 0.0001<br>group; P = 0.3449<br>session x group; P = 0.4925	session; ****<br>group; N.S.<br>session x group; N.S.
		Lidocaine in CA1	17	0	Conditioning; 15.4 ± 1.92%, Reactivation; 45.3 ± 2.94%, Test 1; 49.1 ± 2.95%, Test 2; 19.2 ± 2.29%				
	Motility (pixel / sec.)	Vehicle in CA1	16	0	1-3 min; 82942 ± 5663 pixel, 4-6 min; 52279 ± 2849 pixel, Reactivation; 25391 ± 2195 pixel,<br>Test 1; 20818 ± 1627 pixel, Test 2; 39945 ± 3217 pixel, Test 3; 45339 ± 3250 pixel	Two-way ANOVA with RM	session; $F_{5,155}$ = 87.23<br>group; $F_{1,31}$ = 5.029<br>session x group; $F_{5,155}$ = 6.992	session; P < 0.0001<br>group; P = 0.0322<br>session x group; P < 0.0001	session; ****<br>group; *<br>session x group; ****
		Lidocaine in CA1	17	0	1-3 min; 85902 ± 5914 pixel, 4-6 min; 53217 ± 3324 pixel, Reactivation; 25085 ± 1547 pixel,<br>Test 1; 23383 ± 1916 pixel, Test 2; 39270 ± 3184 pixel, Test 3; 79124 ± 6808 pixel				
5d	Freezing (%)	Vehicle in BLA	13	0	Conditioning; 14.6 ± 2.10%, Reactivation; 49.7 ± 2.94%, Test 1; 50.7 ± 3.29%, Test 2; 22.7 ± 3.04%	Two-way ANOVA with RM	session; $F_{3,72}$ = 105.1<br>group; $F_{1,24}$ = 0.5618<br>session x group; $F_{3,72}$ = 0.8580	session; P < 0.0001<br>group; P = 0.4608<br>session x group; P = 0.4670	session; ****<br>group; N.S.<br>session x group; N.S.
		Lidocaine in BLA	13	0	Conditioning; 13.5 ± 1.72%, Reactivation; 47.9 ± 3.35%, Test 1; 44.5 ± 3.29%, Test 2; 24.0 ± 1.83%				
	Interaction time (sec.)	Vehicle in BLA	13	0	1 min; 15.4 ± 5.04 sec, 2 min; 24.5 ± 4.71 sec, 3 min; 29.7 ± 4.39 sec,<br>4 min; 35.4 ± 2.39 sec, 5 min; 31.7 ± 2.64 sec	Two-way ANOVA with RM	time; $F_{4,120}$ = 4.293<br>group; $F_{1,120}$ = 14.01<br>time x group; $F_{4,120}$ = 1.440	time; P = 0.0028<br>group; P = 0.0003<br>time x group; P = 0.2251	time; **<br>group;***<br>time x group; N.S.
		Lidocaine in BLA	13	0	1 min; 32.5 ± 2.83 sec, 2 min; 35.7 ± 3.17 sec, 3 min; 34.2 ± 2.61 sec,<br>4 min; 40.6 ± 1.72 sec, 5 min; 34.7 ± 3.66 sec				
	Latency to 1st interaction (sec.)	PBS in BLA	13	0	77.6 ± 18.87 sec	Two-tailed unpaired t-test	t_{24} = 3.013	P = 0.0060	**
		Lidocaine in BLA	13	0	20.1 ± 2.97 sec				
6b	Number of c-Fos positive cells BLA	ArchT-Light OFF	9	0	21.3 ± 1.89 cells	One-way ANOVA	$F_{2,29}$ = 11.62	P = 0.0002	***
		ArchT-Light ON	11	0	9.9 ± 1.40 cells				
		EYFP-Light ON	12	0	19.8 ± 2.02 cells				
	Number of c-Fos positive cells ACC	ArchT-Light OFF	9	0	23.2 ± 2.49 cells	One-way ANOVA	$F_{2,29}$ = 13.35	P < 0.0001	****
		ArchT-Light ON	11	0	8.3 ± 1.05 cells				
		EYFP-Light ON	12	0	17.9 ± 2.28 cells				
6d	Number of c-Fos positive cells BLA	ChR2-Light OFF	11	0	6.5 ± 0.73 cells	One-way ANOVA	$F_{2,27}$ = 25.44	P < 0.0001	****
		ChR2-Light ON	12	0	14.9 ± 1.44 cells				
		EYFP-Light ON	7	0	4.0 ± 0.70 cells				
	Number of c-Fos positive cells ACC	ChR2-Light OFF	11	0	5.6 ± 0.66 cells	One-way ANOVA	$F_{2,27}$ = 12.17	P = 0.0002	***
		ChR2-Light ON	12	0	15.8 ± 2.28 cells				
		EYFP-Light ON	7	0	5.9 ± 1.32 cells				
6f	Number of c-Fos positive cells BLA	ArchT-Light OFF	11	4	22.5 ± 1.70 cells	One-way ANOVA	$F_{2,32}$ = 11.88	P = 0.0001	***
		ArchT-Light ON	11	4	12.3 ± 1.24 cells				
		EYFP-Light ON	13	0	19.6 ± 1.47 cells				
	Number of c-Fos positive cells ACC	ArchT-Light OFF	11	4	16.8 ± 2.87 cells	One-way ANOVA	$F_{2,32}$ = 5.096	P = 0.0120	*
		ArchT-Light ON	11	4	7.6 ± 2.23 cells				
		EYFP-Light ON	13	0	16.4 ± 1.71 cells				
7c	Freezing (%)	Light OFF	12	1	Conditioning; 10.0 ± 1.35%, Test; 54.2 ± 3.20%	Two-way ANOVA with RM	session; $F_{1,23}$ = 140.8<br>group; $F_{1,23}$ = 7.873<br>session x group; $F_{2,23}$ = 18.16	session; P < 0.0001<br>group; P = 0.0100<br>session x group; P = 0.0003	session; ****<br>group; *<br>session x group; ***
		Light ON	13	1	Conditioning; 12.2 ± 1.41%, Test; 33.1 ± 4.73%				
7d	Number of c-Fos positive cells in BLA	Light OFF	12	1	29.7 ± 2.16 cell	Two-tailed unpaired t-test	t_{23} = 9.414	P < 0.0001	****
		Light ON	13	1	11.5 ± 1.15 cell				
	Number of c-Fos positive cells in ACC	Light OFF	12	1	44.6 ± 4.13 cell	Two-tailed unpaired t-test	t_{23} = 8.561	P < 0.0001	****
		Light ON	13	1	13.4 ± 1.13 cell				

Supplementary Table 2. Sampling and statistical analysis details in Supplementary Fig. 1, 4-7 and 10.

Fig. #	Category	Group	Sample size (n)		Score	Statistical test	Degree of Freedom & F/t Value	p-value	Significance?
			Exact size (n)	Excluded (n)					
1	Number of Arc+ cells in BLA	Unpaired	3	0	Cyto; 11.9 ± 0.62, Nuc; 8.4 ± 0.91, Cyto/Nuc; 3.7 ± 0.51 cells	Two-tailed unpaired t-test	BLA;<br>Cyto; t4 = 1.799<br>Nuc; t4 = 0.9879<br>Cyto/Nuc; t4 = 5.376	BLA;<br>Cyto; P = 0.1464<br>Nuc; P = 0.3791<br>Cyto/Nuc; P = 0.0058	BLA;<br>Cyto; N.S.<br>Nuc; N.S.<br>Cyto/Nuc; **
		Paired	3	0	Cyto; 15.2 ± 1.75, Nuc; 9.4 ± 0.44, Cyto/Nuc; 7.4 ± 0.48 cells				
	Number of Arc+ cells in CA1	Unpaired	3	0	Cyto; 28.7 ± 2.85, Nuc; 13.0 ± 1.07, Cyto/Nuc; 7.3 ± 0.19 cells		CA1<br>Cyto; t4 = 1.009<br>Nuc; t4 = 2.031<br>Cyto/Nuc; t4 = 12.37	CA1<br>Cyto; P = 0.3701<br>Nuc; P = 0.1121<br>Cyto/Nuc; P = 0.0002	CA1<br>Cyto; N.S.<br>Nuc; N.S.<br>Cyto/Nuc; ***
		Paired	3	0	Cyto; 32.2 ± 2.08, Nuc; 16.7 ± 1.45, Cyto/Nuc; 13.8 ± 0.48 cells				
	Number of Arc+ cells in Entorhinal cortex	Unpaired	3	0	Cyto; 22.6 ± 0.40, Nuc; 11.4 ± 1.09, Cyto/Nuc; 6.4 ± 0.48 cells		Entorhinal cortex<br>Cyto; t4 = 3.044<br>Nuc; t4 = 2.563<br>Cyto/Nuc; t4 = 5.343	Entorhinal cortex<br>Cyto; P = 0.0383<br>Nuc; P = 0.0624<br>Cyto/Nuc; P = 0.0059	Entorhinal cortex<br>Cyto; *<br>Nuc; N.S.<br>Cyto/Nuc; **
		Paired	3	0	Cyto; 27.4 ± 1.56, Nuc; 15.2 ± 0.99, Cyto/Nuc; 13.7 ± 1.26 cells				
	Number of Arc+ cells in Piriform cortex	Unpaired	3	0	Cyto; 11.9 ± 1.16, Nuc; 6.7 ± 0.67, Cyto/Nuc; 3.9 ± 0.73 cells		Piriform cortex<br>Cyto; t4 = 0.9829<br>Nuc; t4 = 1.677<br>Cyto/Nuc; t4 = 2.734	Piriform cortex<br>Cyto; P = 0.3813<br>Nuc; P = 0.1680<br>Cyto/Nuc; P = 0.0522	Piriform cortex<br>Cyto; N.S.<br>Nuc; N.S.<br>Cyto/Nuc; N.S.
		Paired	3	0	Cyto; 13.7 ± 1.39, Nuc; 10.3 ± 2.08, Cyto/Nuc; 6.2 ± 0.44 cells				
	Number of Arc+ cells in Prefrontal cortex	Unpaired	3	0	Cyto; 20.2 ± 1.46, Nuc; 8.4 ± 1.11, Cyto/Nuc; 5.7 ± 1.15 cells		PFC<br>Cyto; t4 = 3.625<br>Nuc; t4 = 2.415<br>Cyto/Nuc; t4 = 4.500	PFC<br>Cyto; P = 0.0223<br>Nuc; P = 0.0731<br>Cyto/Nuc; P = 0.0108	PFC<br>Cyto; *<br>Nuc; N.S.<br>Cyto/Nuc; *
		Paired	3	0	Cyto; 26.7 ± 1.02, Nuc; 13.2 ± 1.64, Cyto/Nuc; 11.7 ± 0.67 cells				
	Number of Arc+ cells in Anterior cingurate cortex	Unpaired	3	0	Cyto; 11.3 ± 0.19, Nuc; 5.8 ± 0.29, Cyto/Nuc; 3.1 ± 0.56 cells		ACC<br>Cyto; t4 = 6.614<br>Nuc; t4 = 2.261<br>Cyto/Nuc; t4 = 6.002	ACC<br>Cyto; P = 0.0027<br>Nuc; P = 0.0866<br>Cyto/Nuc; P = 0.0039	ACC<br>Cyto; **<br>Nuc; N.S.<br>Cyto/Nuc; **
		Paired	3	0	Cyto; 15.2 ± 0.56, Nuc; 7.4 ± 0.68, Cyto/Nuc; 7.0 ± 0.33 cells				
	Number of Arc+ cells in Ectorhinal cortex	Unpaired	3	0	Cyto; 26.2 ± 3.25, Nuc; 11.0 ± 1.26, Cyto/Nuc; 7.7 ± 1.07 cells		Ectorhinal cortex<br>Cyto; t4 = 0.4335<br>Nuc; t4 = 0.5007<br>Cyto/Nuc; t4 = 0.5695	Ectorhinal cortex<br>Cyto; P = 0.6870<br>Nuc; P = 0.6429<br>Cyto/Nuc; P = 0.5995	Ectorhinal cortex<br>Cyto; N.S.<br>Nuc; N.S.<br>Cyto/Nuc; N.S.
		Paired	3	0	Cyto; 28.2 ± 3.28, Nuc; 12.8 ± 3.32, Cyto/Nuc; 9.0 ± 2.08 cells				
	Number of Arc+ cells in Visual cortex	Unpaired	3	0	Cyto; 41.2 ± 5.86, Nuc; 14.3 ± 5.10, Cyto/Nuc; 8.7 ± 2.99 cells		Visual cortex<br>Cyto; t4 = 1.465<br>Nuc; t4 = 1.094<br>Cyto/Nuc; t4 = 2.150	Visual cortex<br>Cyto; P = 0.2168<br>Nuc; P = 0.3354<br>Cyto/Nuc; P = 0.0979	Visual cortex<br>Cyto; N.S.<br>Nuc; N.S.<br>Cyto/Nuc; N.S.
		Paired	3	0	Cyto; 50.0 ± 1.26, Nuc; 20.6 ± 2.51, Cyto/Nuc; 16.0 ± 1.64 cells				
	Number of Arc+ cells in Sensory cortex	Unpaired	3	0	Cyto; 37.0 ± 6.08, Nuc; 11.4 ± 1.56, Cyto/Nuc; 9.1 ± 1.16 cells		Sensory cortex<br>Cyto; t4 = 1.537<br>Nuc; t4 = 1.035<br>Cyto/Nuc; t4 = 1.578	Sensory cortex<br>Cyto; P = 0.1992<br>Nuc; P = 0.3591<br>Cyto/Nuc; P = 0.1897	Sensory cortex<br>Cyto; N.S.<br>Nuc; N.S.<br>Cyto/Nuc; N.S.
		Paired	3	0	Cyto; 46.9 ± 2.12, Nuc; 15.2 ± 3.30, Cyto/Nuc; 13.3 ± 2.41 cells				
	Number of Arc+ cells in Retrosplenial cortex	Unpaired	3	0	Cyto; 22.3 ± 3.67, Nuc; 9.0 ± 1.67, Cyto/Nuc; 7.0 ± 1.50 cells		Retrosplenial cortex<br>Cyto; t4 = 0.5742<br>Nuc; t4 = 1.594<br>Cyto/Nuc; t4 = 1.112	Retrosplenial cortex<br>Cyto; P = 0.5966<br>Nuc; P = 0.1861<br>Cyto/Nuc; P = 0.3285	Retrosplenial cortex<br>Cyto; N.S.<br>Nuc; N.S.<br>Cyto/Nuc; N.S.
		Paired	3	0	Cyto; 25.7 ± 4.50, Nuc; 5.7 ± 1.26, Cyto/Nuc; 4.9 ± 1.16 cells				
4	Zif268 positive cells / DAPI (%)	OFF Dox ArchT-Light OFF	6	0	38.2 ± 2.76%	One-way ANOVA	F3,20 = 15.02	P < 0.0001	****
		OFF Dox ArchT-Light ON	6	0	14.1 ± 2.22%				
		ON Dox ArchT-Light ON	6	0	35.7 ± 4.00%				
		OFF Dox EYFP-Light ON	6	0	42.6 ± 3.78%				
5	Interaction time (sec.)	No-IS	12	0	1 min; 36.4 ± 3.82 sec, 2 min; 31.6 ± 4.19 sec, 3 min; 32.7 ± 3.71 sec,<br>4 min; 31.6 ± 4.50 sec, 5 min; 24.5 ± 4.83 sec	Two-way ANOVA with RM	time; F4,110 = 0.9164<br>group; F1,110 = 15.93<br>time x group; F4,110 = 3.369	time; P = 0.4572<br>group; P = 0.0001<br>time x group; P = 0.0122	time; N.S.<br>group; ***<br>time x group; *
		IS	12	0	1 min; 12.2 ± 4.19 sec, 2 min; 21.1 ± 4.11 sec, 3 min; 27.0 ± 3.73 sec,<br>4 min; 26.1 ± 4.99 sec, 5 min; 25.1 ± 5.56 sec				
	Latency to 1st interaction (sec.)	No-IS	12	0	7.4 ± 1.22 sec	Two-tailed unpaired t-test	t22 = 4.234	P = 0.0003	***
		IS	12	0	446.9 ± 9.25 sec				
6a	Freezing (%)	Vehicle	11	6	Conditioning; 8.3 ± 3.16%, Test; 45.1 ± 5.53%	Two-way ANOVA with RM	session; F1,22 = 57.14<br>group; F1,22 = 13.02<br>session x group; F1,22 = 17.83	session; P < 0.0001<br>group; P = 0.0016<br>session x group; P = 0.0004	session; ****<br>group; **<br>session x group; ***
		Lidocaine	13	7	Conditioning; 7.0 ± 2.52%, Test; 17.4 ± 2.92%				
6b	Motility (pixel/min)	Vehicle	7	1	Conditioning;<br>1 min; 80233 ± 6772 pixel, 2 min; 70457 ± 6243 pixel, 3 min; 71208 ± 7504 pixel,<br>4 min; 65571 ± 7484 pixel, 5 min; 60323 ± 5282 pixel, 6 min; 52383 ± 6142 pixel<br><br>Test;<br>1 min; 62408 ± 8760 pixel, 2 min; 47422 ± 7876 pixel, 3 min; 55392 ± 6786 pixel	Two-way ANOVA with RM	Conditioning session;<br>time; F5,78 = 8.644<br>group; F1,78 = 0.2657<br>time x group; F5,78 = 0.7214<br><br>Test session;<br>time; F2,39 = 2.946<br>group; F1,39 = 1.382<br>time x group; F2,39 = 0.5359	Conditioning session;<br>time; P < 0.0001<br>group; P = 0.6077<br>time x group; P = 0.6094<br><br>Test session;<br>time; P = 0.0643<br>group; P = 0.2469<br>time x group; P = 0.5894	Conditioning session;<br>time; ****<br>group; N.S.<br>time x group; N.S.<br><br>Test session;<br>time; N.S.<br>group; N.S.<br>time x group; N.S.
		Lidocaine	8	1	Conditioning;<br>1 min; 89938 ± 6206 pixel, 2 min; 80387 ± 5205 pixel, 3 min; 75371 ± 4348 pixel,<br>4 min; 59187 ± 6464 pixel, 5 min; 55224 ± 5323 pixel, 6 min; 50793 ± 4843 pixel<br><br>Test;<br>1 min; 59146 ± 4422 pixel, 2 min; 46064 ± 6044 pixel, 3 min; 41967 ± 3082 pixel				
		Vehicle-No IS	8	1	1 min; 23.4 ± 4.53 sec, 2 min; 25.7 ± 4.69 sec, 3 min; 31.1 ± 3.36 sec,<br>4 min; 35.2 ± 2.85 sec, 5 min; 30.6 ± 3.72 sec				

6c	Interaction time (sec.)	Vehicle-IS	8	2	1 min; 7.8 ± 4.00 sec, 2 min; 18.8 ± 5.49 sec, 3 min; 30.3 ± 3.06 sec, 4 min; 26.7 ± 3.74 sec, 5 min; 29.5 ± 4.13 sec	Two-way ANOVA with RM	time; $F_{4,88}$ = 14.80<br>group; $F_{2,22}$ = 1.508<br>time x group; $F_{8,88}$ = 1.347	time; P < 0.0001<br>group; P = 0.2434<br>time x group; P = 0.2314	time; ****<br>group; N.S.<br>time x group; N.S.
		Lidocaine-IS	9	2	1 min; 8.6 ± 3.29 sec, 2 min; 23.7 ± 4.57 sec, 3 min; 25.4 ± 3.80 sec, 4 min; 31.3 ± 5.29 sec, 5 min; 33.2 ± 3.63 sec				
	Latency to 1st interaction (sec.)	Vehicle-No IS	8	1	27.5 ± 10.29 sec	One-way ANOVA	$F_{2,22}$ = 4.626	P = 0.0210	*
		Vehicle-IS	8	2	80.4 ± 16.36 sec				
		Lidocaine-IS	9	2	78.3 ± 13.83 sec				
7	c-Fos positive cells / DAPI (%)	OFF Dox ChR2-Light OFF	6	0	0.3 ± 0.19%	One-way ANOVA	$F_{3,20}$ = 779.7	P < 0.0001	****
		OFF Dox ChR2-Light ON	6	0	20.0 ± 0.58%				
		ON Dox ChR2-Light ON	6	0	0.1 ± 0.09%				
		OFF Dox EYFP-Light ON	6	0	0.6 ± 0.34%				
10	Zif268 positive cells / DAPI (%)	OFF Dox Light OFF	6	0	13.5 ± 1.25%	One-way ANOVA	$F_{2,15}$ = 54.14	P < 0.0001	****
		OFF Dox Light ON	6	0	2.0 ± 0.21%				
		ON Dox Light ON	6	0	10.7 ± 0.63%				
