# Supplementary material for: A cortical cell ensemble in the posterior parietal cortex controls past experience-dependent memory updating
Source: Nat Commun. 2022 Jan 11;13:41. doi: 10.1038/s41467-021-27763-x (PMC8752845; doi:10.1038/s41467-021-27763-x)
Supplement: Supplementary file 3 — Reporting Summary [file 41467_2021_27763_MOESM3_ESM.pdf]

nature portfolio

Corresponding author(s): Kaoru Inokuchi

Last updated by author(s): Nov 15, 2021

Reporting Summary

Nature Portfolio wishes to improve the reproducibility of the work that we publish. This form provides structure for consistency and transparency in reporting. For further information on Nature Portfolio policies, see our Editorial Policies and the Editorial Policy Checklist.

Statistics

For all statistical analyses, confirm that the following items are present in the figure legend, table legend, main text, or Methods section.

n/a	Confirmed	
☐	☒	The exact sample size (*n*) for each experimental group/condition, given as a discrete number and unit of measurement
☐	☒	A statement on whether measurements were taken from distinct samples or whether the same sample was measured repeatedly
☐	☒	The statistical test(s) used AND whether they are one- or two-sided<br>*Only common tests should be described solely by name; describe more complex techniques in the Methods section.*
☒	☐	A description of all covariates tested
☐	☒	A description of any assumptions or corrections, such as tests of normality and adjustment for multiple comparisons
☐	☒	A full description of the statistical parameters including central tendency (e.g. means) or other basic estimates (e.g. regression coefficient) AND variation (e.g. standard deviation) or associated estimates of uncertainty (e.g. confidence intervals)
☐	☒	For null hypothesis testing, the test statistic (e.g. *F*, *t*, *r*) with confidence intervals, effect sizes, degrees of freedom and *P* value noted<br>*Give P values as exact values whenever suitable.*
☒	☐	For Bayesian analysis, information on the choice of priors and Markov chain Monte Carlo settings
☒	☐	For hierarchical and complex designs, identification of the appropriate level for tests and full reporting of outcomes
☒	☐	Estimates of effect sizes (e.g. Cohen's *d*, Pearson's *r*), indicating how they were calculated

Our web collection on statistics for biologists contains articles on many of the points above.

Software and code

Policy information about availability of computer code

Data collection	Freezing and motility data were collected with video tracking system, CompACT VAS/DV (Muromachi Kikai).<br>Image acquisition from target regions was performed with LSM780 confocal microscopy (Zeiss) with ZEN2.3 SP1 software (Zeiss).
Data analysis	The statistical analysis was performed with GraphPad Prism version 6.

For manuscripts utilizing custom algorithms or software that are central to the research but not yet described in published literature, software must be made available to editors and reviewers. We strongly encourage code deposition in a community repository (e.g. GitHub). See the Nature Portfolio guidelines for submitting code & software for further information.

Data

Policy information about availability of data

All manuscripts must include a data availability statement. This statement should provide the following information, where applicable:

- Accession codes, unique identifiers, or web links for publicly available datasets
- A description of any restrictions on data availability
- For clinical datasets or third party data, please ensure that the statement adheres to our policy

The data generated in this study are provided in the Supplementary Information/Source Data files. Source data are provided with this paper. Further information will be available from the corresponding author on reasonable request.

Field-specific reporting

Please select the one below that is the best fit for your research. If you are not sure, read the appropriate sections before making your selection.

☒ Life sciences ☐ Behavioural & social sciences ☐ Ecological, evolutionary & environmental sciences

For a reference copy of the document with all sections, see nature.com/documents/nr-reporting-summary-flat.pdf

Life sciences study design

All studies must disclose on these points even when the disclosure is negative.

Sample size	No statistical methods were used to predetermine sample sizes, which were based on work in previous publications (Ohkawa et al. Cell Reports 11, 261-269, 2015; Nomoto et al. Nature Communications 7, 12319, 2016; Yokose et al. Science 355, 398-403, 2017).
Data exclusions	Mice in which no opsin or Rhodamine B expression was confirmed were excluded from each experiment (See supplementary table 1 and 2).
Replication	Independent animals were used for all studies as replicates. All experiments were repeated at least two times independently with similar results.
Randomization	Mice were randomly assigned to each groups, and experiments were run by alternating between each groups.
Blinding	All experiments were conducted in a blind manner, without any information about the identity of the injected virus or drug, or the specific manipulation for the transgene being expressed.

Reporting for specific materials, systems and methods

We require information from authors about some types of materials, experimental systems and methods used in many studies. Here, indicate whether each material, system or method listed is relevant to your study. If you are not sure if a list item applies to your research, read the appropriate section before selecting a response.

Materials & experimental systems

n/a	Involved in the study	
☐	☒	Antibodies
☒	☐	Eukaryotic cell lines
☒	☐	Palaeontology and archaeology
☐	☒	Animals and other organisms
☒	☐	Human research participants
☒	☐	Clinical data
☒	☐	Dual use research of concern

Methods

n/a	Involved in the study	
☒	☐	ChIP-seq
☒	☐	Flow cytometry
☒	☐	MRI-based neuroimaging

Antibodies

Antibodies used	Primary Antibodies: rabbit anti-GFP (1:1000, Invitrogen, Carlsbad, CA, USA, A11122), goat anti-c-Fos (1:1000, SantaCruz, Santa Cruz, CA, USA, SC-52G), chicken anti-GFP (1:1000, abcam, Cambridge, UK, ab13970), rabbit anti-Egr-1 (1:1000, SantaCruz, Santa Cruz, CA, USA, SC-189).<br>Secondary antibodies: donkey anti-rabbit IgG-Alexa Fluor 488 (1:300, Life Technologies, Carlsbad, CA, USA, A21206), donkey anti-goat IgG-Alexa Fluor 546 (1:300, Life Technologies, Carlsbad, CA, USA, A11056), donkey anti-chicken IgG-Alexa Fluor 488 (1:300, Jackson ImmunoResearch Laboratories, Inc, West Grove, PA, USA, 703-545-155), donkey anti-rabbit IgG-Alexa Fluor 546 (1:300, Life Technologies, Carlsbad, CA, USA, A10040).
Validation	Each antibody is commercially available. The specificity of these antibodies was validated by the manufacturers.<br>Validation profiles for each antibody can be found in the links provided.<br><br>rabbit anti-GFP (Invitrogen, Carlsbad, CA, USA, A11122: https://www.thermofisher.com/antibody/product/GFP-Antibody-Polyclonal/A-11122).<br>goat anti-c-Fos (SantaCruz, Santa Cruz, CA, USA, SC-52G: https://www.scbt.com/p/c-fos-antibody-4?productCanUrl=c-fos-antibody-4&_requestid=3193752).<br>chicken anti-GFP (abcam, Cambridge, UK, ab13970: https://www.abcam.com/gfp-antibody-ab13970.html).<br>rabbit anti-Egr-1 (SantaCruz, Santa Cruz, CA, USA, SC-189: https://www.scbt.com/p/egr-1-antibody-c-19?productCanUrl=egr-1-antibody-c-19&_requestid=3192345).<br>donkey anti-rabbit IgG-Alexa Fluor 488 (Life Technologies, Carlsbad, CA, USA, A21206: https://www.thermofisher.com/antibody/product/Donkey-anti-Rabbit-IgG-H-L-Highly-Cross-Adsorbed-Secondary-Antibody-Polyclonal/A-21206).<br>donkey anti-goat IgG-Alexa Fluor 546 (Life Technologies, Carlsbad, CA, USA, A11056: https://www.thermofisher.com/antibody/product/Donkey-anti-Goat-IgG-H-L-Cross-Adsorbed-Secondary-Antibody-Polyclonal/A-11056).

donkey anti-chicken IgG-Alexa Fluor 488 (Jackson ImmunoResearch Laboratories, Inc, West Grove, PA, USA, 703-545-155: https://www.jacksonimmuno.com/catalog/products/703-545-155).
donkey anti-rabbit IgG-Alexa Fluor 546 (Life Technologies, Carlsbad, CA, USA, A10040: https://www.thermofisher.com/antibody/product/Donkey-anti-Rabbit-IgG-H-L-Highly-Cross-Adsorbed-Secondary-Antibody-Polyclonal/A10040).

Animals and other organisms

Policy information about studies involving animals; ARRIVE guidelines recommended for reporting animal research

Laboratory animals	Naïve male C57BL/6J (Japan SLC, Inc., Shizuoka, Japan) and c-fos-tTA transgenic mice (Mutant Mouse Regional Resource Centre, stock number: 031756-MU) aged 10-18 weeks were used for experiments.<br>c-fos-tTA transgenic mice were raised beginning when they were fetuses on food containing 40 mg/kg Dox and maintained on Dox pellets, except for the labeling day. All mice were maintained on a 12 h light/dark cycle (lights on 8:00 am – 8:00 pm) at 24 ± 3°C and 55 ± 5% humidity, had ad libitum access to food and water, and were housed in a cage with littermates until surgery.
Wild animals	This study did not involve wild animals.
Field-collected samples	This study did not involve samples collected from the field.
Ethics oversight	All animal procedures were approved by the Animal Care and Use Committee of the University of Toyama.

Note that full information on the approval of the study protocol must also be provided in the manuscript.
